# Fatty acid intakes in healthy adults quantified using a food frequency questionnaire compared with red blood cell membrane fatty acid content: A validation study

**DOI:** 10.1111/1747-0080.70039

**Published:** 2025-09-21

**Authors:** Erin D. Clarke, Mitch Duncan, Lisa G. Wood, Jessica J. A. Ferguson, Clare E. Collins

**Affiliations:** ^1^ School of Health Sciences, College of Health, Medicine and Wellbeing University of Newcastle Callaghan New South Wales Australia; ^2^ Food and Nutrition Research Program Hunter Medical Research Institute New Lambton Heights New South Wales Australia; ^3^ School of Medicine and Public Health, College of Health, Medicine and Wellbeing University of Newcastle Callaghan New South Wales Australia; ^4^ Active Living and Learning Research Program Hunter Medical Research Institute New Lambton New South Wales Australia; ^5^ School of Biomedical Sciences and Pharmacy, College of Health, Medicine and Wellbeing University of Newcastle Callaghan New South Wales Australia

**Keywords:** biomarkers, diet records, dietary intake, fatty acids, validation study

## Abstract

**Aims:**

Red blood cell membrane fatty acids can be used alongside self‐reported dietary assessment methods, such as food frequency questionnaires, to measure the validity of self‐reported intakes. This study aimed to validate fatty acid intakes estimated from the Australian Eating Survey food frequency questionnaire against red blood cell profiles of healthy Australian adults.

**Methods:**

Demographic data and dietary intakes of fatty acids were collected, and red blood cell membrane fatty acid composition was measured using gas chromatography. Bland–Altman plots and concordance correlation coefficients (*ρ*_c_) examined levels of agreement between measures and were adjusted for confounders.

**Results:**

A total of 58 participants (67% female, mean age 39.6 years) yielded 105 observations. Total saturated, total monounsaturated, total polyunsaturated fats including Eicosapentaenoic acid, Docosahexaenoic acid, Docosapentaenoic acid, and linoleic acid were within limits of agreement with moderate associations (*ρ*_c_ = 0.26–0.59). All adjusted models weakened associations; except total saturated fat retained moderate association in all models (*ρ*_c_ = 0.24–0.58). Omega‐3 supplement use weakened associations for all fatty acids, except saturated and total omega‐3 polyunsaturated fats. Carbohydrate intake had the least impact on associations.

**Conclusion:**

Self‐reported intakes from the Australian Eating Survey food frequency questionnaire were in moderate agreement (*ρ*_c_ = 0.20–0.60) with red blood cell membrane fatty acids. This questionnaire may be used as an indicator of self‐reported long‐term dietary fatty acid intake in Australian adults, with caveats for individuals reporting extreme intakes and consideration for evaluating the effects of endogenous synthesis of fatty acids. Future studies are warranted to replicate findings and strengthen translation to other sub‐populations.

## 1 INTRODUCTION

The intake of total dietary fat and different fat types consumed has varying effects on plasma lipids, cardiovascular health, and risk of coronary events.^1^, ^2^ Understanding the proportion and types of dietary fats consumed is important for dietary recommendations for the prevention and management of diet‐related chronic disease.^3^, ^4^ Different types of dietary fats have divergent associations with chronic disease. Higher mortality from all causes, cancer, and cardiovascular disease (CVD) has been shown to be associated with dietary patterns high in saturated fats.^3^ Conversely, dietary patterns high in polyunsaturated fats (PUFA) and monounsaturated fatty acids (MUFA) have been associated with lower mortality from all causes, CVD, and cancer,^3^, ^4^ with linoleic acid, omega‐6 (n‐6) PUFA, and marine‐derived omega‐3 (n‐3) PUFA intake as key drivers of inverse associations with mortality.^4^ These observations among those demonstrated in intervention studies underpin current dietary recommendations around the globe aimed at replacing saturated and trans fats with healthier unsaturated fats.

Long‐term, or usual dietary intake, is commonly assessed using food frequency questionnaires (FFQs), which are a practical method due to ease of administration, low participant burden, and being independent of the need for trained interviewers and hence economical to use.^5^ FFQs collect data on usual intake of a defined list of foods and beverages over a specified period and can provide detailed insight into participants food choices and long‐term dietary habits. However, limitations include that data is self‐reported, commonly semi‐quantitative, and prone to mis‐reporting bias.^6^ Underreporting of less healthy foods, such as those containing large amounts of total and/or saturated fat, has been identified previously^7^, ^8^ along with dietary fat intake identified as at risk of being mis‐reported.^7^ It is important that dietary assessment instruments are validated against objective food biomarkers to identify whether self‐reported intake is reflective of actual dietary intake.

A biological biomarker such as red blood cell (RBC) membrane fatty acids as an objective measure of usual fatty acids intake used alongside a self‐reported dietary assessment method like an FFQ may allow for a greater understanding of dietary fatty acids intake, increasing accuracy and usefulness within validation studies implementing self‐reported intakes.^9^ The concentrations of fatty acids in biological samples such as RBC membranes, which have a half‐life of 120 days,^10^ reflect intake over this time period and have been used alongside self‐reported dietary assessment methods as a biomarker of dietary fat intake to provide further insight into consumption of foods containing essential fatty acids.^11^, ^12^, ^13^, ^14^ RBC membrane fatty acid concentrations have been used previously alongside dietary fatty acids intakes estimated using the cardiovascular^11^ and child^12^ versions of the Australian Eating Survey FFQ, but not against intakes reported by healthy Australian adults using the FFQ version 2011–13.^12^, ^15^ The earlier version of the FFQ used the nutrition database AUSNUT 1999, while the current version uses the current nutrition database (AUSNUT 2011–13). Therefore, the aim was to conduct a validation study comparing fatty acids intakes derived using self‐reported dietary intakes from the FFQ version 2011–13 against RBC membrane fatty acid concentrations in healthy adults residing in NSW, Australia.

## 2 METHODS

Australian adults ≥18 years in the Newcastle region, NSW, Australia were recruited through various local print and web media between September 2019 and March 2020. Participants were screened using an online survey completed via Qualtrics XM System (Provo, Utah, USA). Eligible volunteers had to be weight stable (±4 kg) over the previous 2 months, have internet access, and be able to travel to The University of Newcastle (NSW, Australia) to attend in‐person data collection sessions. Exclusion criteria have been reported elsewhere.^16^ Ethics approval was obtained from The University of Newcastle Human Research Ethics Committee (Approval No. H‐2019‐0147) and informed written consent was obtained from all participants. The trial was registered with the Australian New Zealand Clinical Trials Registry (ANZCTR‐12619001415190). Reporting was guided by the STROBE‐Nut guidelines (Supplement 1).^17^

Demographic information was collected through online surveys conducted using Qualtrics XM System (Provo, Utah, USA): age, sex, ethnicity, education level, marital status, employment status, supplements, and medications. At baseline and follow‐up appointments at 3‐, 6‐, and 9‐months, anthropometric measures included height (cm), weight (kg) and body fat percentage measured using bioelectrical impedance analysis (Inbody 270, Seoul, Korea). Waist circumference (cm) was taken at the narrowest point of the abdomen between the lower costal (10th rib) border and the top of the iliac crest, perpendicular to the long axis of the trunk. All anthropometric measurements were taken twice unless they were out by >0.1 cm (height and waist circumference) or >0.1 kg (weight) in which case, a third measure was taken.

Usual dietary intake over the previous 3 months was assessed using the 120‐item semi‐quantitative FFQ^15^ with 15 additional demographic questions, using the web‐based Qualtrics XM System (Provo, Utah, USA). The FFQ was completed online at baseline, 3‐, 6‐ and 9‐months data collection times. Frequency of intake options ranged from ‘Never’ to ‘4 or more times per day’ for each item. Nutrient intakes, including fatty acids intakes for each item were computed using data from the latest nutritional surveys.^18^, ^19^ Fatty acids per 100 g were available from AUSNUT 2011–13 and dietary fatty acids intake was calculated from the FFQ as used previously.^11^

Venous blood samples were collected from participants after an overnight fast (~10 h) into EDTA tubes by a trained phlebotomist at an accredited pathology service (NSW Health Pathology). Samples were analysed by the pathology service on a VP auto‐analyser using standardised reagents for plasma lipids and insulin. RBC fractions were aliquoted and stored at −80°C until analysis. RBC fractions were transported on dry ice to the Hunter Medical Research Institute for quantification of RBC membrane fatty acids.

RBC membrane fatty acids preparation and determination was conducted using previously established methods.^20^, ^21^, ^22^ RBCs were thawed, lysed, and membranes solubilised and purified according to previously established methods.^21^ This process involved adding 12 mL of hypotonic tris buffer and 12 mL of 0.25 M glucose solution to 500 μL of RBCs. Samples were left to stand on ice for 5 min before the solution was then centrifuged at 10,000 rpm at 4°C for 10 min. The supernatant was collected and discarded. This process was repeated twice more, with the centrifuge set at 12,000 rpm at 4°C for 10 minutes and then at 15,000 rpm at 4°C for 20 min. The pellet was resuspended in 250 μL of glucose solution and 250 μL of tris buffer solution and stored at −80°C prior to analysis.

Total RBC membrane fatty acids were determined using the method established by Lepage and Roy.^20^ A mixture of methanol/toluene (4:1 v/v) containing C13:0 and C19:0 as internal standards and BHT (0.12 g/L) was added to the membrane suspension. To methylate the fatty acids, acetyl chloride was added dropwise while vortexing; samples were then capped and heated at 100°C for 1 h. The sample was cooled, then 6% K_2_CO_3_ was added to stop the reaction. The sample was centrifuged at 3000 rpm at 4°C for 10 min to separate the layers, and the upper toluene layer was used for gas chromatography (GC) analysis of the fatty acid methyl esters. Analysis was undertaken using a 30 m × 0.25 mm (DB‐225) fused carbon‐silica column coated with cyanopropylphenyl (J & W Scientific, Folsom, CA, USA). Fatty acid methyl ester peaks in the samples were identified by comparing their retention times with those of a standard mixture of FA methyl esters (GLC‐462, Nu‐Chek Prep Inc. Elysian, MN, USA) and quantified using a Hewlett Packard 6890 Series Gas Chromatograph with a flame ionisation detector and Chemstations software (version A.04.02, Hewlett Packard, Palo Alto, CA, USA). Helium was used as carrier gas and nitrogen as make‐up gas. The injection port temperature was 200°C, and the detector was 250°C. The column temperature was held at 80°C for 5 min and, in a stepwise fashion, reached a plateau of 220°C. While the following individual RBC membrane fatty acids were quantified, results for all fatty acids may not be reported. The individual fatty acids quantified included C10:0, C12:0, C12:1, C14:0, C14:1n‐7, C18:0, C18:1n‐9 (OLEATE), C18:1n‐7 (VACCENATE), C18:2n‐6, C18:3n‐6 (GAMMA), C18:3n‐3 (ALPHA), C20:0, C20:1n‐9, C20:2n‐6, C20:3n‐6 (HOMO), C20:4n‐6, C20:3n‐3 (DH‐14‐17), C20:5n‐3 (EPA), C22:0, C22:1n‐9, C22:2n‐6, C22:4n‐6, C22:5n‐3 (DPA), C22:6n‐3 (DHA), C24:0, and C24:1n‐9.

Total fatty acids content was the sum of saturated fatty acids (SFA), MUFA, and PUFA for both FFQ and RBC membrane fatty acids. The quantities of individual RBC membrane fatty acids were expressed as the percentage of total RBC membrane fatty acids. The FFQ fatty acid intake was converted to a percentage of total fatty acids, where total fatty acids was the sum of SFA, PUFA, and MUFA.

The level of agreement between fatty acids measures from the FFQ and RBC was assessed using Bland–Altman plots and the Concordance Correlation Coefficient (CCC)^23^, ^24^ using R (version 4.4.0).^25^ Due to the repeated measures, both the Bland–Altman plots and the CCC were estimated using a mixed model approach^26^ using lme4.^27^ Bland–Altman plots were produced using ggplot2^28^ and estimated using a mixed model, using the difference between the two measures as the response, a fixed effect for group, and a random intercept for individuals. The mixed models conducted to estimate the CCC used the fatty acids derived from each measure (i.e., FFQ vs. RBC) as the response and included a fixed effect for each measure (FFQ vs. RBC), group, and a random intercept for individuals.^26^ For Bland–Altman plots, measures of fatty acids between the FFQ and RBC membranes were measured in different units (i.e., g/day vs. %); therefore, plots were examined using either percentage differences, log transformed values, or standardised values.^26^, ^29^, ^30^ The normality of the differences was visually inspected using histograms and Q–Q plots, and the transformation method that resulted in the most reasonable distribution was used in the analysis. Plots using standardised values will not show fixed bias by design and can be interpreted based on the magnitude of differences across the average values.^30^ CCC was estimated using raw, log transformed, or standardised values, and model residual plots were used to assess model assumptions and the most reasonable fitting model selected. All outcomes were standardised except that linolenic acid was log transformed. The percentile‐based 95% confidence intervals for the mean difference and the limits of agreement (LOA) in the Bland–Altman plots, and the 95% confidence interval for the CCC were estimated using bootstrapping with 1000 replications using the Boot package.^31^, ^32^ CCC values were interpreted as follows: <0.20 poor, 0.20–0.60 moderate, and >0.6 strong as per previous studies.^33^, ^34^, ^35^

To examine the potential impact of a priori defined confounders, the CCC were also estimated when adjusting for age, sex, body mass index (BMI), energy intake, carbohydrate intake, and n‐3 PUFA supplement use in four different models, which included a fixed effect for group and a random intercept for individual. Due to high collinearity, models did not include carbohydrate and energy in the same model. To examine the potential impacts of endogenously derived fatty acids palmitic acid (16:0) and stearic acid (18:0), a sensitivity analysis was conducted by omitting these two fatty acids from the summation of total RBC derived SFA.

## 3 RESULTS

Data collection occurred during three‐monthly rounds repeated over 9 months, with some post‐baseline blood collection appointments disrupted by COVID‐19. In total, 105 observations from 58 participants had complete data and were included for analysis. Of the 58 participants, 17 participants had complete data for two timepoints and 15 for three timepoints between 3‐ and 9‐months. Participant baseline demographics are presented in Table 1. The majority were female (67%), non‐smoking (93%) with an average age of 39.6 (SD 15.4) years, BMI in the overweight category,^36^ fasting total cholesterol, and blood glucose concentrations in the normal/healthy range.^37^, ^38^ Dietary intakes and RBC membrane fatty acids are presented in Table 2. Mean daily intakes were 8358 kJ (SD 2704 kJ), 77.3 g (SD 27.1 g) fat, 34.1% total energy from fat, and 28 g (SD 12.7 g) combined saturated plus trans‐fat intake (12.6% total energy).

**TABLE 1:** Baseline demographics of participants (*n* = 58).

	Mean, *N*	(SD), %
Age (years)	39.6	(15.4)
Sex		
Male	19	32.8
Female	39	67.2
Height (cm)	169.8	(8.5)
Weight (kg)	75.4	(18.1)
BMI (kg/m^2^)	26.1	(5.6)
BIA—SMM—Score	29.3	(6.9)
BIA—FAT—Score	21.4	(9.3)
BIA—VFA—Score	9.7	(5.3)
Marlowe‐Crowne Social Desirability Scale (MCSDS) (0–13)	5.2	(1.6)
No. medications/supplements used	1.5	(2.1)
Education categories		
Highschool/no formal education	18	31.0
Trade/diploma	10	17.2
University	30	51.7
Employment categories		
Full time (≥35 h/week)	23	39.7
Part time (<35 h/week)/casual	20	34.5
Student, not employed, prefer not to say	15	25.9
Smoking status		
No	54	93.1
Yes	4	6.9
Cholesterol (mmol/L)	4.9	(1.1)
HDL‐cholesterol (mmol/L)	1.6	(0.4)
LDL‐cholesterol (mmol/L)	2.8	(1.0)
Non‐HDL cholesterol (mmol/L)	3.3	(1.1)
Total Chol:HDL Ratio	3.2	(1.0)
Triglycerides (mmol/L)	1.0	(0.6)
Glucose (mmol/L)	4.7	(0.5)
Insulin (mlU/L)	7.1	(4.1)

Abbreviations: AES, Australian Eating Survey; BIA, bioelectrical impedance analysis; HDL, high‐density lipoprotein; LDL, low‐density lipoprotein; SMM, skeletal muscle mass; VFA, visceral fat area.

**TABLE 2:** Dietary intakes and red blood cell fatty acids (*n* = 58).

	Mean, *N*	(SD), %
AES dietary intake		
Total energy intake (kJ/day)	8358.3	(2704.2)
Protein (g)	81.8	(27.4)
Fat (g/days)	77.3	(27.1)
Saturated fat (g/day)	26.9	(12.1)
Trans fat (mg)	1118.6	(599.7)
Monounsaturated fat (g/day)	32.3	(10.8)
Polyunsaturated fat (g/day)	11.4	(3.7)
Alpha Linolenic acid (18:3‐n3) (g)	1.1	(0.4)
Total long chain omega‐3 polyunsaturated fat (mg)	260.1	(205.1)
Eicosapentanoic acid (20:5‐n3) (mg)	79.7	(67.3)
Docosapentaenoic acid (22:5‐n3) (mg)	55.3	(30.9)
Docosahexaenoic acid (22:6‐n3) (mg)	125.2	(115.7)
Linoleic acid (18:2‐n6) (g)	9.7	(3.3)
Carbohydrate (g/day)	214.6	(76.8)
Sugar (g/day)	100.4	(44.2)
Fibre (g)	30.5	(11.4)
Sodium (mg)	1589.6	(566.9)
Cholesterol (mg)	255.4	(140.7)
Alcohol (g)	8.6	(10.7)
Red blood cell fatty acids, % of total fatty acids^a^		
Total saturated fat	43.1	(1.5)
Total monounsaturated fat	18.3	(1.6)
Total polyunsaturated fat	38.6	(1.5)
Total long chain omega‐3 polyunsaturated fat	8.6	(1.9)
Eicosapentaenoic acid (20:5‐n3)	1.0	(0.6)
Docosapentaenoic acid (22:5‐n3)	2.3	(0.5)
Docosahexaenoic acid (22:6‐n3)	5.2	(1.2)
Linoleic acid (18:2‐n6)	9.9	(1.4)

Abbreviation: AES, Australian Eating Survey.

^a^ Data for red blood cell fatty acids are referring to methyl esters and are presented as a proportion of total red blood cell fatty acids. Data for alpha‐linolenic acid is not presented because levels for this fatty acid were undetected in this sample.

**FIGURE 1:**
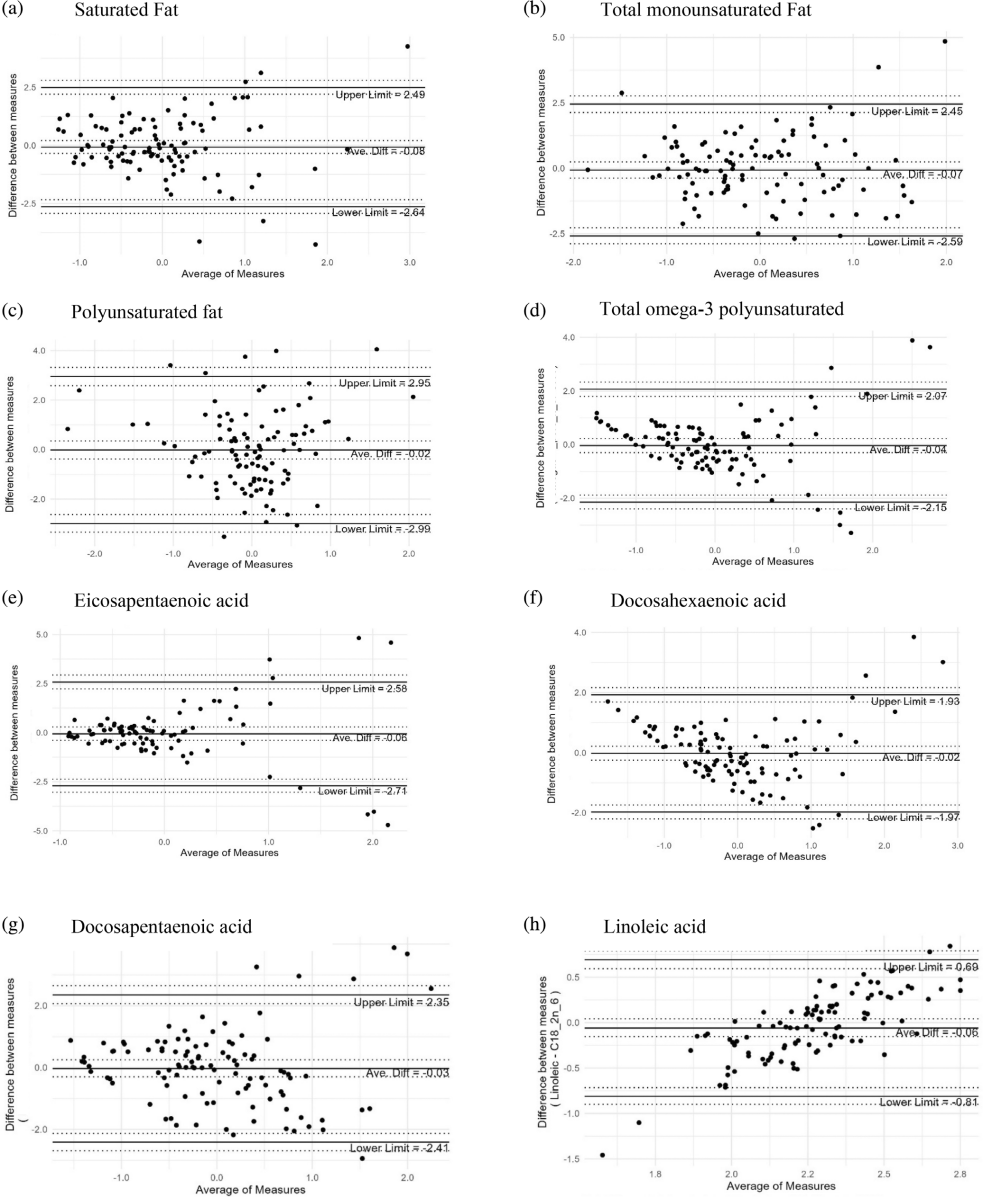
Bland–Altman plots with limits of agreement and 95% confidence intervals (indicated by dotted lines). Data presented are standardised mean values for self‐reported dietary fats from the food frequency questionnaire and red blood cell derived fatty acids.

The LOA between self‐reported dietary fatty acids intake and RBC membrane fatty acids composition is presented in Bland–Altman plots, Figure 1. The majority of values across all fatty acids were within the respective LOA, with only some values falling outside these limits. For SFA, there was a tendency towards greater differences between measures at higher values. MUFA values appeared consistent across the range, with very few values falling outside the LOA. Most values for PUFA appeared central around zero for the average of measures; although at lower average values, there was some tendency towards overestimation of self‐reported dietary fatty acids intake. The Bland–Altman plot for total n‐3 PUFA presents some funnelling, indicating a tendency towards overestimating at lower average values and both greater over‐and underestimation at higher average values. Most Eicosapentaenoic acid (EPA) values were lower and indicated low bias, with most values close to the mean of the differences; however, greater bias at higher average outcomes was indicated by slight funnelling. The FFQ tended to overestimate Docosahexaenoic acid (DHA) values at lower average values; additionally, the FFQ tended to both over‐ and underestimate values at higher average outcomes. Docosapentaenoic acid (DPA) values appeared mostly consistent across the range of average values, with a tendency towards greater differences between the measures at higher values. The FFQ appears to underestimate values at lower average values and overestimate values at higher average values for linoleic acid.

Table 3 presents CCC's for the agreement between fatty acids derived from the FFQ and RBC membranes, with higher positive values indicating better agreement. All outcomes displayed statistically significant moderate levels of agreement (crude CCC, 0.28–0.59) aligning with Bland–Altman plots. LOAs were borderline strong for linoleic acid (CCC 0.59, 95% CI 0.42, 0.76) and DHA (CCC 0.59, 95% CI 0.45, 0.73). No outcomes displayed a strong or weak LOA.

**TABLE 3:** Unadjusted and adjusted concordance correlation coefficients expressing agreement between fatty acids derived from the food frequency questionnaire and fatty acids derived from red blood cells (*n* = 105).

	CCC (95% CI)				
Outcome	Crude	Model 1	Model 2	Model 3	Model 4
Total SFA	0.42 (0.27, 0.59)	0.55 (0.42, 0.69)	0.24 (0.14, 0.41)	0.58 (0.45, 0.71)	0.29 (0.17, 0.46)
Total SFA:Sensitivity	0.43 (0.28, 0.60)	0.50 (0.36, 0.64)	0.18 (0.09, 0.35)	0.52 (0.39, 0.67)	0.23 (0.11, 0.42)
Total MUFA	0.52 (0.38, 0.67)	0.21 (0.08, 0.37)	0.20 (0.08, 0.35)	0.27 (0.12, 0.42)	0.24 (0.10, 0.40)
Total PUFA	0.28 (0.10, 0.46)	0.38 (0.21, 0.59)	0.14 (0.05, 0.32)	0.38 (0.22, 0.59)	0.17 (0.06, 0.37)
Linoleic acid	0.59 (0.42, 0.76)	0.16 (0.05, 0.32)	0.15 (0.05, 0.31)	0.28 (0.14, 0.44)	0.27 (0.14, 0.44)
Total long chain n‐3 PUFA	0.56 (0.41, 0.72)	0.33 (0.20, 0.50)	0.26 (0.13, 0.46)	0.40 (0.27, 0.53)	0.36 (0.22, 0.51)
Eicosapentaenoic	0.43 (0.24, 0.68)	0.20 (0.08, 0.37)	0.17 (0.07, 0.32)	0.26 (0.14, 0.41)	0.20 (0.09, 0.33)
Docosapentaenoic	0.49 (0.36, 0.65)	0.53 (0.39, 0.67)	0.20 (0.10, 0.37)	0.55 (0.41, 0.69)	0.24 (0.12, 0.42)
Docosahexaenoic	0.59 (0.45, 0.73)	0.17 (0.04, 0.32)	0.16 (0.04, 0.32)	0.21 (0.06, 0.36)	0.19 (0.04, 0.34)

*Note*: Concordance correlation coefficient estimates along with their confidence intervals are presented for each variable. Total saturated fat: Sensitivity encompasses red blood cell derived total saturated fat for which palmitic acid (16:0) and stearic acid (18:0) have been omitted from the calculation of this fatty acid subgroup in order to account for the potential influence of their endogenous synthesis from carbohydrates.^53^, ^54^, ^55^ Confidence intervals are bootstrapped percentile based on 1000 replications. Estimates of Concordance Correlation Coefficient are based on Parker et al.^26^ Model 1 includes fixed effect for group and measurement (AES vs. RBC), age, sex, BMI and energy and random intercept for individual. Model 2 includes fixed effect for group and measurement (AES vs. RBC), age, sex, BMI and energy, omega‐3 supplement use and random intercept for individual. Model 3 includes fixed effect for group and measurement (AES vs. RBC), age, sex, BMI and carbohydrate and random intercept for individual. Model 4 includes fixed effect for group and measurement (AES vs. RBC), age, sex, BMI and carbohydrate, omega‐3 supplement use and random intercept for individual. All values are standardised prior to analysis except Linoleic Acid and Polyunsaturated Fat which were log transformed.

Abbreviations: CCC, concordance correlation coefficient; LC n‐3PUFA, long‐chain omega‐3 polyunsaturated fatty acids; MUFA, monounsaturated fatty acids; PUFA, polyunsaturated fatty acids; SFA, saturated fatty acids.

When adjusting for potential confounders (Table 3), SFA was most robust to the influence of confounders, with values retaining moderate CCCs. Associations weakened, indicative of reduced CCCs for MUFA, linoleic acid, total n‐3 PUFA, EPA and DHA in all adjusted models. Adjusting for n‐3 PUFA supplement use appeared to have the greatest impact, resulting in weak associations for all fatty acids except SFA and total n‐3 PUFA. Carbohydrate intake did not appear to impact associations, with all fatty acids retaining moderate CCCs (CCC 0.21–0.58). When n‐3 PUFA supplement use and carbohydrate intake were both accounted for, only PUFA, EPA, and DHA associations weakened. Adjusted CCC values for PUFA were higher compared to unadjusted values in all models except for the two models where n‐3 PUFA supplement use was accounted for. No adjusted model for any fatty acid achieved a strong level of agreement.

## 4 DISCUSSION

Consistent statistically significant relationships were identified between diet and RBC membrane fatty acids for SFA, MUFA, total n‐3 PUFA, and DPA. Whereas PUFA, linoleic acid, EPA, and DHA were less consistent, especially at more extreme intakes. These findings provide evidence to support the validity of the FFQ in quantifying dietary fatty acid intakes and support its use for assessing self‐reported fatty acid intakes in Australian adults. These findings support the use of selected RBC membrane fatty acids as previously reported biomarkers of long‐term dietary fat intake.^10^, ^39^

Demographic characteristics of the current study population had some similarities and differences with the general Australian population, with more females than males in the current study. Mean BMI (26 kg/m^2^) was comparable to the Australian population where almost two‐thirds are overweight or obese.^40^ Total dietary fat intake was moderate‐high, with percentage total energy from fat (34.1%) reaching the upper end of the Acceptable Macronutrient Distribution Range (20%–35% of total energy). Combined saturated and trans‐fat intake exceeded the recommended 10% of total energy (12.4%).^41^ These intakes are slightly lower than proportions of total fat (39.1%) and saturated/trans‐fat (15.9%) reported in the 2022–2023 Australian apparent consumption data.^42^

Dietary intake of linoleic acid^43^ and both dietary intake and RBC membrane proportions of SFA, MUFA, and PUFA were similar to previous reports in Australian adults.^44^ This includes our previous FFQ validation in similarly aged adults (mean age 37 years) from regional NSW.^16^ Daily intakes of PUFA, EPA, DPA, and DHA were also comparable to previous reports in healthy relatively young (mean age 35 years) adults^45^ and older adults (mean age 49.5 years).^35^ When compared to the intake of healthy Australian women (18–35 years), values in the current study were lower for n‐3 PUFAs, EPA, and DHA, and RBC membrane total n‐3 PUFAs and DPA. However, the composition of RBC membrane EPA and DHA was comparable (other fatty acids were not summarised).^46^ Fatty acids derived from the FFQ and RBC membranes were within LOA for all fatty acid subclasses (SFA, MUFA, and PUFA) including total n‐3 PUFA, EPA, DHA, and DPA. While most MUFA, PUFA, and DPA values were consistent across the range of average values, extreme values for DHA, EPA, and linoleic acid were less consistent, indicating some over‐ and/or underestimation at extremes of dietary fatty acids intake. Adjusted models weakened as indicated by reduced CCCs. This has been demonstrated in previous studies,^11^, ^47^ indicating associations between diet‐RBC fatty acids may not be independent of diet and lifestyle factors.

SFA retained a moderate agreement between diet‐RBC membrane measures in all adjusted models (CCC 0.24–0.58), with agreement strengthened when energy and carbohydrate intake were independently accounted for in addition to age, sex, and BMI. This contrasts previous validation studies reporting no or weak associations between SFA derived from the diet versus biospecimen (RBC membrane, plasma phospholipids, and plasma).^10^, ^35^, ^48^ This may be expected since SFA and MUFA classes are endogenously synthesised from carbohydrates,^49^, ^50^ and thus influence RBC membrane fatty acid composition. SFA's palmitic (16:0) and stearic acid (18:0), while influenced by dietary SFA intake, are also synthesised endogenously via hepatic de novo lipogenesis,^51^, ^52^ which is also impacted by dietary intake of carbohydrates and fats.^53^, ^54^, ^55^ Omission of these two SFAs in a sensitivity analysis demonstrated little impact on LOA for SFA in the current study, with slight improvements in two adjusted models accounting for total energy‐ and carbohydrate intake. The moderate‐high fat dietary intake (~34% energy) of the current sample may explain why the LOA for SFAs was stronger than other studies, as lipogenesis has been shown to be down regulated and lower in individuals consuming diets higher in fat (>30% energy).^56^, ^57^ This suggests that the FFQ remains a robust indicator of self‐reported dietary SFA and MUFA intakes among Australians. Interpretation of SFA derived from the FFQ may require caution in populations consuming lower fat diets, and verification in these sub‐populations could be explored in future studies.

The agreement between diet‐derived RBC membrane values for total n‐3 PUFA also remained moderate and robust in all adjusted models (CCC 0.26–0.56) which is consistent with previous studies demonstrating moderate to strong associations for total PUFA.^10^, ^11^, ^35^ These observations were expected as n‐3 PUFAs (EPA, DPA, and DHA) are largely exogenous in origin, derived predominately from intake of fatty fish and/or fish oil supplements.^58^, ^59^, ^60^ Findings from the current study support the validation of the FFQ for dietary intake assessment of total n‐3 PUFAs. However, caution may be required when assessing individual n‐3 PUFAs, as associations between diet‐derived RBC membrane values for EPA and DHA weakened after adjustment in all models (except when adjusting for carbohydrate intake), with the weakest association evident in models accounting for n‐3 PUFA supplement use. Similar observations have been reported in healthy Swiss adults,^61^ whereas in other studies reporting stronger agreements, the number of individuals consuming n‐3 PUFA/fish oil supplements was proportionally very small,^10^, ^35^ or adjustment for their use did not alter correlation coefficients,^62^ Due to reporting limitations, the current study did not quantify amounts of n‐3 PUFA consumed as supplements. However, these observations underscore the importance of collecting information on dietary supplement use concurrently with the FFQ when quantifying dietary fatty acids intakes.

Few studies have examined the comparative validity of dietary fatty acid intakes estimated by a semi‐quantitative FFQ relative to RBC membrane fatty acid concentrations in Australian adult populations. A key strength of the current study is the range of fatty acids presented, which is broader than typically found in previous dietary intake validation studies, which tend to only focus on n‐3 PUFAs.^13^, ^14^, ^62^, ^63^ This study also expands on the current literature by demonstrating novel moderate associations between diet‐derived RBC membrane values for SFA and MUFAs in the context of a higher‐fat diet, which further supports dietary intake data for SFA and MUFA derived from self‐reported FFQ in individuals with higher fat intake. The comparability of the current sample's characteristics with the Australian population, including dietary intake of various fatty acids, is also a strength of this study, supporting external validity and informing future study design in other sub‐populations or regions within Australia.

Limitations of the current study include disruptions to data collection due to COVID‐19, which resulted in some missing data. Limitations inherent to RBC membrane fatty acids as a biomarker of total fat intake need to be acknowledged. First, α‐linolenic acid was not present in sufficient quantities for detection in RBC membranes, which could be attributed to several mechanisms, including a large proportion of dietary α‐linolenic acid being oxidised, as interconversion of n‐3 PUFAs in humans is limited.^64^ Therefore, dietary α‐linolenic acid estimated via the FFQ could not be validated against RBC membrane fatty acids. Secondly, the composition of RBC membrane fatty acids is not proportional to the amounts consumed from the diet. For example, arachidonic acid makes up a larger proportion of RBC membranes than that quantified by dietary assessment methods.^65^, ^66^ This is because some fatty acids are more readily altered by diet, such as n‐3 fatty acids, whereas SFAs, like stearic acid (C18:0), are more tightly controlled and less influenced by dietary intake.^67^ Thirdly, endogenous synthesis of fatty acids may influence the concentration of RBC membrane fatty acids, contributing to discrepancies when comparing dietary‐derived fatty acid intake to RBC membrane content. While this has led to the use of a combination of biospecimens in some studies, for example, RBC, plasma phospholipids, and cholesterol esters,^68^ using multiple lipid biomarkers is not always feasible and can be both costly and burdensome compared to fatty acids intake derived through dietary assessment methods.^9^, ^69^ Lastly, agreement was assessed in the Bland–Altman plots using either percentage differences, log transformed values, or standardised values. Although this is an established way to deal with measurements made in different units and proportional bias,^26^, ^29^, ^30^ the plots are presented in different units from the original measures and need to be considered when interpreting these figures.

The current study identified that self‐reported intakes from the Australian Eating Survey FFQ were significantly associated with RBC membrane fatty acid concentrations, which indicates long‐term dietary fatty acid intake. These findings are comparable to those using an earlier version of the FFQ and confirm that the current version of the FFQ can be used as a low‐cost, low‐burden, non‐invasive measure of self‐reported dietary fatty acid intakes in the general Australian adult population. Future research is required in larger samples to further understand potential impacts of the FFQ to over‐ and/or underestimate dietary fatty acids in extreme intake cases, such as populations where dietary fat intake may be low.

## AUTHOR CONTRIBUTIONS

*Conceptualization*: C.E.C. *Methodology*: C.E.C. *Formal analysis*: M.D. *Writing—original draft preparation*: E.D.C. *Writing—review and editing*; M.D., L.W., J.J.A.F., and C.E.C. *Supervision*; L.W., C.E.C. *Project administration*: E.D.C. All authors have read and agreed to the published version of the manuscript.

## CONFLICT OF INTEREST STATEMENT

The validated food frequency and diet quality score derived from it have been developed by C.E.C and research team, usage incurs a cost. J.J.A.F holds a separate, part‐time position with the Sanitarium Health Food Company who had no input into the research. All other authors declare no conflicts of interest.

## ETHICS STATEMENT

Ethics approval was obtained from The University of Newcastle Human Research Ethics Committee (Approval No. H‐2019‐0147) and informed written consent obtained from all participants.

## Supporting information


**Data S1:** Supporting Information

## Data Availability

The data that support the findings of this study are available on request from the corresponding author. The data are not publicly available due to privacy or ethical restrictions.
